# Upadacitinib treatment of refractory livedoid vasculopathy: a case report and literature review^؆^

**DOI:** 10.1016/j.abd.2025.501236

**Published:** 2025-11-04

**Authors:** Jiecheng Zheng, Xiaoqian Liang, Xueyi Huang, Jia Liao

**Affiliations:** Department of Dermatology, Zhongshan Second People's Hospital, Zhongshan City, Guangdong Province, China

*Dear Editor,*

Livedoid vasculopathy (LV) is a rare, chronic, recurrent vascular disease that manifests as erythema, purpuric macules, painful ulcers, livedo reticularis, atrophic porcelain-white scars of the lower extremities, typically in ankles and feet. Although the pathophysiological process is not yet fully elucidated, thrombosis of the dermal vessels appears to be a key event.^1^ The conventional therapies consist of anticoagulants, antiplatelets, corticosteroids, immunosuppressive agents, thrombolytics,^2^ etc. However, patients commonly encounter disease relapse. Upadacitinib, a relatively new JAK1 inhibitor, has never been reported to be used in treating LV in the retrieved literature. Herein, we report a case of refractory LV resistant to conventional therapy which exhibited great efficacy following treatment with Upadacitinib.

A 30-year-old female presented with over 5-year history of painful, ulcerative lesions of the feet and ankles. Physical examination showed multiple purpura maculae, painful ulcerations with small, dilated blood vessels, as well as sporadic porcelain-white scars (Fig. 1). Laboratory investigations were found with positive anti-cardiolipin antibody, and helped us rule out other LV-associated systemic disorders. A skin biopsy revealed ulceration formation, vascular dilation, swollen vessel walls with fibrinoid necrosis, erythrocyte exosmosis, as well as infiltration of perivascular lymphocytes and neutrophils (Fig. 2). The examination findings and clinical manifestations were strongly consistent with the diagnosis of LV. Although the patient was previously prescribed rivaroxaban, aspirin, mycophenolate morphenate, and oral corticosteroids (prednisone 30 mg once daily), she was still confronted with disease relapse. We initiated Upadacitinib at a daily dose of 15 mg orally, along with topical ointment, hirudoid (mucopolysaccharide polysulfate cream). The patient manifested significant improvement in erythema and pain within 10-days, and achieved nearly complete resolution of ulceration and remained only post-inflammatory hyperpigmentation within 6-weeks (Fig. 3). No adverse events were observed during the 20-week follow-up. For clinical assessment, the expected time for Upadacitinib maintenance is 6-months, so as to reduce the recurrence of the patient.

**Fig. 1:**
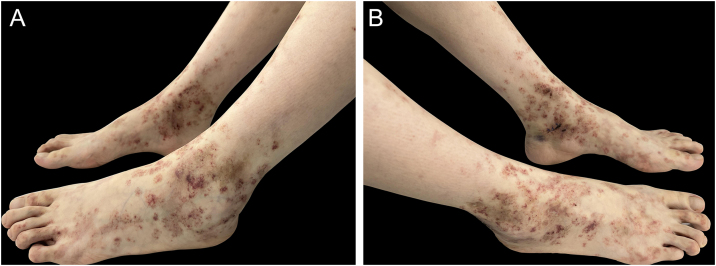
Physical examination showed multiple purpura macula, painful ulcerations with small dilated blood vessels as well as sporadic porcelain-white scars.

**Fig. 2:**
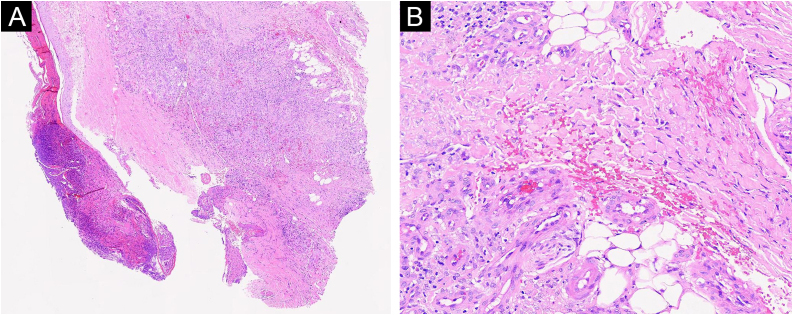
Pathological and immunohistochemical specimens. (A) Low-power view showed ulceration formation, increased vascular proliferation and dilation (Hematoxylin & eosin, ×50). (B) The infiltration of perivascular lymphocytes and swollen vessel walls with fibrinoid necrosis, erythrocyte exosmosis (original magnification: ×400).

**Fig. 3:**
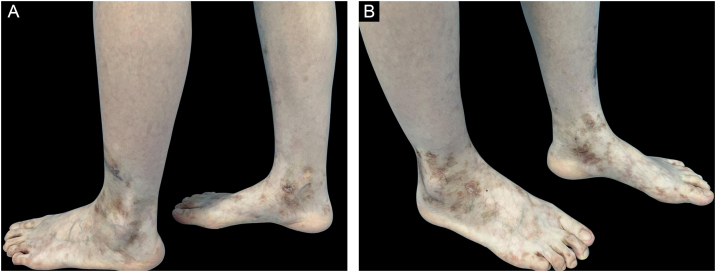
After 6-weeks of treatment with Upadacitinib, the patient manifested significant improvement in erythema and pain, achieved nearly complete resolution of ulceration and remained only hyperpigmentation.

The Janus Kinase (JAK) and Signal Transduction Transcription Activator (STAT) signal transduction pathways interplay with a wide range of immunocytes and over 50 cytokines. It was reported JAK-STAT pathways have enhanced activity in vascular endothelial cells;^3^ moreover, JAK signaling could significantly affect inflammation, coagulation, and thrombosis formation.^4^ The overactivation of JAK/STAT pathways induces immune imbalance and vascular damage.

LV is a thrombo-occlusive vascular disorder, which on histopathology manifests as hyaline thrombosis, fibrin deposits in vessel walls, along with perivascular focal lymphocyte infiltration. As described previously, it is still difficult to treat and prone to relapse. Existing experience suggests that steroids were suggested to be effective even as a monotherapy, and the traditional anti-inflammatory drugs could successfully improve the clinical outcomes. Concurrently, employing colchicine and prednisolone as monotherapy could exhibit higher efficacy than using pentoxifylline and aspirin alone in LV patients, indicating an indispensable role of inflammation in LV pathogenesis.^5^ Given its pathophysiology, JAK inhibitors should theoretically play an important role in preventing the flares and recurrence of LV.

Previous small-scale retrospective studies and case reports have shown considerable efficacy when utilizing JAK inhibitors in LV treatment,^5^, ^6^ as summarized in Table 1. Han et al. observed 8 patients who received 2 mg/day of baricitinib for treating refractory LV, and found that all enrolled patients experienced a significant regression with a mean remission time of 7.75-weeks. Similarly, in the case series of Song et al., 3 patients with LV were prescribed 2 mg/day of baricitinib. One patient obtained darkening and reduced erythema by month-6, and the other two obtained complete ulcer healing, respectively, by month-1 and month-2. Regarding other JAK inhibitors, Chen et al. reported the rapid improvement in a 31-year-old female developing LV after using abrocitinib 100 mg once daily. In addition, Jia et al. administered tofacitinib 5 mg twice per day on a 17-year-old male with refractory LV, the ulcers were completely healed within a month with no recurrence during the 56-week follow-up. The aforementioned clinical practice suggests that JAK inhibitors are promising therapeutic options to treat LV. Upadacitinib is an oral, small-molecule JAK1 inhibitor that has been approved for multiple inflammatory diseases in dermatology. There are no reports to date for its application on LV. In our case, using 15 mg/day of Upadacitinib, the patient achieved great improvement within 10-days and the ulceration completely healed within 6-weeks.

**Table 1:** Overview of JAK inhibitors as a novel treatment for livedoid vasculopathy.

Study	No.	Sex	Age	Duration of Disease	Previous Treatment	Treatment	Outcome	Adverse event	Follow-up time
Han et al.	8	5F/3M	8∼36	7∼120 months	Diverse therapies (Aspirin, corticosteroid; thalidomide; tripterygium glycosides; rivaroxaban; enoxaparin; compound glycyrrhizin; Chinese traditional anti-inflammatory drugs)	Baricitinib 2 mg/day	All experienced significant regression; remission times ranging from 3- to 13-weeks, with a mean remission time of 7.75 ± 3.45 weeks	None	11‒28 weeks
Chen et al.	1	1F	31	2-years	NR	Abrocitinib 100 mg/day	Complete remission was achieved after 6-weeks, with only post-inflammatory hyperpigmentation remaining	None	12-weeks
Song et al.	3	1F/2M	8‒26	6∼72 months	Diverse therapies (Prednisone, rivaroxaban, thalidomide, aspirin)	Baricitinib 2 mg/day	Patient 1: reduced erythema by month-6; Patient 2/3: Complete healing of ulcer by month 1/2	None	
Jia et al.	1	1M	17	3-years	Colchicine, thalidomide, dipyridamole, rivaroxaban and aspirin	Tofacitinib 5 mg twice daily	Complete healing of ulcer by month-1	None	56-weeks

JAK, Janus Kinase; M, Male; F, Female; NR, Not Report.

To our knowledge, this is the first report in which Upadacitinib was utilized to treat LV. Further studies should be conducted to confirm its efficacy and long-term safety.

## ORCID IDs

Xiaoqian Liang: 0000-0001-8806-8533; Xueyi Huang: 0000-0002-3114-589X

## Authors’ contributions

Jiecheng Zheng: The study concept and design; data collection, or analysis and interpretation of data; Writing of the manuscript or critical review of important intellectual content; Data collection, analysis and interpretation.

Xiaoqian Liang: The study concept and design; data collection, or analysis and interpretation of data; Writing of the manuscript or critical review of important intellectual content; Data collection, analysis and interpretation.

Xueyi Huang: Data collection, or analysis and interpretation of data.

Jia Liao: Effective participation in the research guidance; Final approval of the final version of the manuscript.

## Financial support

This research received no specific grant from any funding agency in the public, commercial or not-for-profit sectors.

## Research data availability

Does not apply.

## Conflicts of interest

None declared.
